# Solar-driven seawater desalination *via* plasmonic hybrid MOF/polymer and its antibacterial activity†

**DOI:** 10.1039/d3ra02242k

**Published:** 2023-06-20

**Authors:** Ola. R. Hayes, Amr Awad Ibrahim, Mina Shawky Adly, S. E. Samra, A. M. A. Ouf, S. A. El-Hakam, Awad I. Ahmed

**Affiliations:** a Chemistry Department, Faculty of Science, Mansoura University Al-Mansoura 35516 Egypt amr_awad@mans.edu.eg

## Abstract

In recent years, solar seawater desalination has been considered to be a promising and cost-effective technique to produce clean sources for water treatment and water deficiency. In addition, this technique shows high photothermal conversion efficiency by solar collectors to transfer solar energy into heat and the transformation of molecules in the capillaries of solar evaporators. In this study, we report the preparation of graphene-supported MIL-125 with polyurethane foam (MGPU) for solar steam generation. We modified MGPU by using the plasmonic nanoparticles of Ag and a polymer of polyaniline to increase the evaporation rate. Polyurethane foam can float on the surface of water and self-pump water by its hydrophilic porous structure, superior thermal insulation capabilities, and easy fabrication. MIL-125 has a high salt rejection and higher water permeability. It can reduce the affinity between water molecules and the pore surface of membrane, making it simple for water molecules to move through the pores. GO is a great alternative for steam generation applications since it exhibits broad-band light. The strong solar absorption, photothermal conversion efficiency, and photoreaction efficiency are enhanced by the use of silver nanoparticles in the photoreaction. The salt resistance capability is enhanced in saline water in the presence of polyaniline in a composite. Under one solar irradiation, the Ag/PANI/GO@MIL-125 (Ag-PMG) nanocomposite demonstrates an average 1.26 kg m^2^ h^−1^ rate of evaporation and an efficiency as high as 90%. The composite exhibits remarkable stability and durability after more than 10 cycles of use without a noticeable decrease in activity. In addition, the composite exhibits excellent organic dye removal from contaminated water and generates pure condensed freshwater. The antibacterial photoactivity of the photocatalysts was examined against *B. subtilis* and *E. coli*. The results demonstrate that Ag-PMG shows higher antibacterial activity than MIL-125 and PMG. It was shown that the presence of rGO, PANI, and Ag in the sample enhances the antimicrobial activity.

## Abstract 2

In recent years, solar seawater desalination has been considered to be a promising and cost-effective technique to produce clean sources for water treatment and water deficiency.

## Graphical abstract

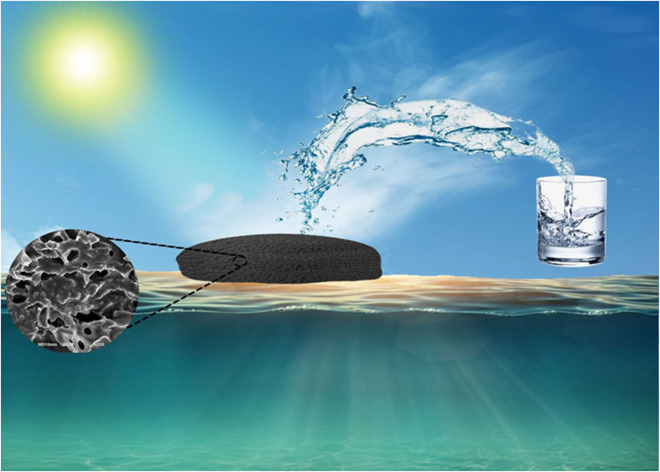

## 1. Introduction

Freshwater supplies are currently being depleted due to high wastewater discharge and industry usage.^1^ Currently, the decrease in pure water is one of the important global problems that threaten humanity and needs to be solved.^2^ Pure water is necessary for different uses, including drinking, agriculture, and industrial uses.^3^ According to the United Nations Environmental Program (UNEP), one-third of the world's population lives in water-stressed areas, and two-thirds of the global population will endure water scarcity by 2025.^4^ The population and energy needs will continue to rise, accompanied by economic expansion. By 2035, global energy consumption is expected to rise by 31%.^5^ Desalination is one technique for extracting pure water from salty, brackish, or contaminated water. All desalination techniques can be used to resolve the freshwater source shortages. These processes, which include multi-effect desalination (MED), reverse osmosis (RO), desalination by humidification and dehumidification (HD), multi-stage flash distillation (MSF), membrane distillation (MD), mechanical vapour compression (MVC) desalination, and low-temperature desalination (LTD), frequently involve high energy consumption, high material requirements, and high costs.^6–8^ We can improve the evaporation efficiency and use alternative energy sources for heating in seawater desalination, such as solar energy.^9,10^ The most renewable energy source is solar energy, which has large technical feasible potential (about 60 TW) among all sources of renewable energy. The solar steam generation strategies can easily transform light into heat water with low photothermal conversion efficiency because of the significant heat loss.^11^ Three categories of using solar energy are currently available, such as photothermal conversion (PTC), photoelectric conversion,^12^ and photochemical conversion.^13^ For heat insulation and liquid supply, a substrate and high-efficiency PTC materials are required.^14^

Photothermal conversion materials (PCMs) are cost-effective and simple methods to generate steam that floats at the water–air interface, which may produce heat from solar energy. Additionally, the PCMs can produce solar steam and freshwater, localize heat in the upper region, and decrease heat loss to the bulk water. It is necessary to produce photothermal conversion materials with small thermal conductivity, self-floating properties and low cost.^15–22^ Light absorption by the photothermal layer is important for large-efficiency solar desalination. Plasmonic metals for surface plasmon resonance (SPR), such as gold,^23^ silver,^24^ and alumina,^25^ can be used for efficiently heating water by increasing the temperatures over its boiling point, and can absorb more than 95% of the sunlight for solar desalination. We demonstrate other solar absorbers with surface plasmon resonance to enhance the rate of evaporation with metal–organic frameworks, graphene oxide and polymer.

Metal–organic frameworks (MOFs) are important candidates because of their high porosity, charged surface, hydrophilicity and evaporation-driven electricity generation, such as MIL-125 for efficient water desalination. Titanium-based MOFs have high porosity, structural stability, and high electrical and optical properties, nontoxicity, and are low-cost. Their photocatalytic applications include H_2_ production, CO_2_ reduction, H_2_O_2_ production, organic and inorganic waste remediation, and N_2_ fixation.^26–30^ Lately, the use of metal–organic frameworks (MOFs) as new materials during water desalination has attracted much attention among engineers. MIL-125(Ti) has light response characteristics due to the development of new Ti-MIL structures and their application in many fields.^31^ The organic ligands in MOFs can be stimulated by light and transport electrons to the metal center, making them useful as semiconductor-like photocatalysts.^32^ MIL-125 has excellent efficiency, water permeability and salt rejection in the desalination process.^33–36^ MIL-125 and its modification can be used in photocatalytic processes.^37–45^ Titanium MOFs have important research applications as anti-inflammatory, antibacterial and anticancer agents.

For solar desalination, graphene oxide (GO) has also been described as a photothermal material due to its effective light absorption, highly controlled microstructures, and production simplicity. GO is a great candidate for steam generation systems, including optoelectronic and biological applications, since it exhibits a broad spectrum of light absorption from the visible to NIR region. By the size effect, graphene oxide (GO) helps to reject hydrated salt ions.^46,47^ Graphene oxide (GO) and rGO are necessary for toxicity to Gram-positive and Gram-negative bacteria.^46^ Polyaniline (PANI) is known for its flexibility, high conductivity, good light absorption property, chemical stability, and solar-heating interfacial evaporation.^49^ PANI is used in many fields, including electrostatic discharge systems, batteries, and chemical and biochemical sensors. PANI has high antibacterial activity because of the electrostatic interaction between PANI and bacteria, and the increase in the rate of the host materials for catalysis and antibacterial activity.^50,51^ The Ag and PANI materials have shown antibacterial activity against *B. subtilis* bacteria, and has also been reported against another bacteria.^52^

Herein, we report the fabrication of nanostructured MIL-125 as solar absorbers because of its large solar absorptance, and cost-effective synthesis. To enhance the evaporation performance, we report a solar thermal evaporator composite of plasmatic GO-based MIL-125. The conductivity, thermal stability and light absorption property were enhanced by modification using PANI and Ag. In this paper, we prepared a floating polyurethane foam (PU), which is mixed with a photothermal composite by a simple method.

## 2. Experimental

### 2.1. Materials and chemicals

All solvents and chemicals were supplied and used without purification. Titanium(iv) isopropoxide (TTIP), 1,4-benzene dicarboxylic acid (H_2_BDC), *N*,*N*-dimethylformamide (DMF) and methanol were acquired from Sigma Aldrich. For the synthesis of GO, the following materials were used as follows: graphite powder (99.95%), potassium permanganate (KMnO_4_), sodium nitrate (NaNO_3_), sulfuric acid (H_2_SO_4_), and 30% hydrogen peroxide (H_2_O_2_). Hydrochloric acid (HCl), absolute ethanol silver nitrate (AgNO_3_), sodium borohydride, ammonium persulfate and aniline were used for surface modification.

### 2.2. Synthesis of graphene oxide (GO)

Graphite flakes were used to prepare graphene oxide by using a modified Hummer's technique. In a 250 mL beaker, 115 mL of concentrated H_2_SO_4_ was added at 0 °C (in an ice bath). A mass of 2.5 g ground NaNO_3_ was added to the previous solution, then 4.0 g graphite powder was added after all of the ground NaNO_3_ was completely dissolved. After a duration of 20 min, 15.0 g KMnO_4_ was added to the mixture very slowly, ensuring that the temperature did not exceed 5 °C. After 15 min, the heat of the mixture reached 35 °C in 3 h and transfered to 2 L beaker, followed by the addition of 230 mL DI H_2_O. A volume of 700 mL DI H_2_O was added after 20 min at a temperature of 80 °C. A volume of 20 mL 10% H_2_O_2_ was poured into the mixture after 20 min, and the color changed to golden yellow. The resulting graphene oxide was washed with 2 L acidified water (HNO_3_), followed by 5 L hot DI H_2_O (80–100 °C) at 1 L each time. The brown emulsion was put into a convection oven at 60 °C for drying.

### 2.3. Synthesis of MIL-125

By using the solvothermal method, we synthesized MIL-125 depending on the following procedures. 1,4-Benzene dicarboxylic acid (15 mmol) was dissolved in a mixture of methanol (5 mL) and anhydrous DMF (45 mL). Subsequently, TTIP (9 mmol) was added to the mixture and stirred for 10 min. The mixture was placed into a Teflon tube and heated at 120 °C for 16 hours, and put in a conventional oven. The autoclave was cooled to reach room temperature. Finally, we collected the white MIL-125 by using a centrifuge, and washed the precipitate several times with DMF and then diluted ethanol. Finally, the white solid was dried at 80 °C, and maintained in a small glass vial.

### 2.4. Synthesis of MIL-125@rGO composites

The same previous procedures were applied, but the calculated amount of GO (0.4 g) was dispersed in DMF for 30 min and added into 1,4-benzene dicarboxylic acid (15 mmol). The products were then dissolved in a mixture of methanol (5 mL) and anhydrous DMF (45 mL). Subsequently, TTIP (9 mmol) was added to the mixture with stirring for 10 min. The reaction mixture was placed into a Teflon tube and heated at 120 °C for 16 h in a conventional oven. The autoclave was cooled to room temperature. Finally, the white precipitate was collected by using a centrifuge, and was washed several times with DMF and diluted ethanol. Finally, the white solid was dried at 80 °C and maintained in a small glass vial.

### 2.5. Synthesis of Ag-doped MIL-125@GO

MIL-125@GO (1.0 g) was suspended in 20 mL DI water, and then (5%) Ag nanoparticles were incorporated. Subsequently, a calculated amount of AgNO_3_ was dissolved in DI water, added to the previous mixture, and then stirred overnight. Silver ions were reduced using NaBH_4_, and the final product was centrifuged and dried at 80 °C.

### 2.6. Synthesis of PANI-MIL-125@GO nanocomposite

For the synthesis of PANI-MIL-125@GO, 1.0 g of GO/MIL125 was ultrasonically dispersed into 0.4 mL of aniline stirred in 80 mL HCl (1 M), and stirred for 30 min. Typically, ammonium persulfate (0.91 g, 0.04 mmol) was added to the previous mixture, and stirred for 3 h in an ice bath. The resulting green powder was collected by centrifuge, and washed several times with DI water, then dried at 80 °C overnight.^53^

### 2.7. Synthesis of Ag/PANI-MIL-125@GO nanocomposite

For the synthesis of Ag/PANI/GO/MIL125, 1.0 g of PANI-MIL-125@GO was suspended in 10 mL DI water, added to a certain amount of AgNO_3_ and stirred overnight. Silver ions were reduced using NaBH_4_, and the final product was centrifuged and dried at 80 °C. The prepared samples were labeled as follows M, MG, PMG, Ag-MG and Ag-PMG for MIL-125, MIL-125@GO, PANI/MIL-125@GO, Ag/MIL-125@GO and Ag/PANI/MIL-125@GO, respectively.

### 2.8. Characterization techniques

XRD measurements of all prepared samples were recorded by a Philips (PW 150) X-ray diffractometer instrument using Ni-filtered Cu K_α_ radiation with wavelength (*λ*) = 1.540 Å at 45 mA and 40 kV. The scanning was performed in a two-theta angle from 1° to 70° with a step time of 0.5 seconds and a step size of 0.02°. The peaks were indexed and used in conjunction with Bragg's law. FT-IR analysis of all prepared samples was accomplished by the *in situ* FT-IR spectroscopic technique on Nicolet Magna-IR 550 spectrometers with 128 scans and 4 cm^−1^ resolution in the mid-IR region of 400–4000 cm^−1^. Each sample was mixed with KBr substance and pressed into a thin wafer that was placed into the IR cell, and then the spectrum was recorded. All prepared samples were analyzed by using the SEM technique to study the structure morphology and the chemical composition. The surface images of the prepared nano-scaled samples were acquired using a JEOL-JSM-6510 LV scanning electron microscope. Avery small quantity of the prepared samples was placed on a gold-coated grid, and scanned with a focused electrons beam. X-ray photoelectron spectroscopy gives information about the chemical state, the elemental composition and the electronic state of the elements in the samples, and is a surface-sensitive quantitative spectroscopic technique. For X-rays of the wavelength Al Kα, the energy of the photon is known to be 1486.7 eV. Nitrogen adsorption/desorption measurements were performed to determine the specific surface area and pore volume of the as-fabricated catalysts, and were performed at a liquid nitrogen temperature (−196 °C). A Sci-Sun-300 AAA solar simulator was used to display the solar steam generation tests, and the solar power was adjusted using a power meter. A thermocouple was used to obtain each thermal absorber surface's temperature throughout the desalination process, and an IR camera recorded the surfaces' thermal pictures. The ion concentrations of desalinated water and saline, as well as dyes in solution, were measured by an inductively coupled plasma (ICP) mass spectrometer and a UV-vis spectrophotometer, respectively.

### 2.9. Synthesis of the photoabsorber materials

We fabricated a one-layer system for solar-driven water desalination by using polyurethane foam. Firstly, 0.5 g of the synthesized composites were mixed with 3 mL of polyol, and then 2 mL of diisocyanate liquid for 6–8 s. After 10 min, the foam was cut into disks with 1 cm thickness and 4.5 cm diameter, and kept for further use. When polyols are added in excess, the final PU seems to be hydrophilic and softer. Isocyanates are very reactive and are necessary for the curing process, the rigid part of the polyurethane and hydrophobic polyurethanes. Finally, polyurethane foam was floated in 100 mL of saline water.

### 2.10. Evaporation performance under one sun

Using a solar simulator, the steam generation test took place for 60 minutes at a temperature of 22 °C ± 3 °C and 50% humidity. The illumination power of the solar simulator was 1 sun, which is equivalent to 1 kW m^−2^. In a 100 mL beaker of DI water or saline water, polyurethane foam was floated. The mass loss of water at a certain time was measured by an electronic analytical balance every 5 minutes in evaporation. An IR camera was used to show the temperature change on the surface of the foams both before and after radiation. The evaporation rate was calculated using eqn (1).1
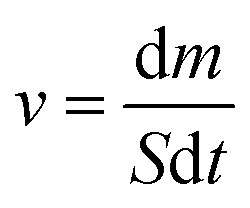


The evaporation rate (*ν*) can be measured by eqn (1), where *S* is the surface area of the polyurethane foam, *m* is the water evaporation's mass loss (kg), and *t* is the time of illumination.2
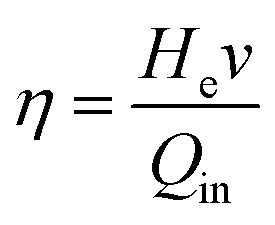
which *n* is the rate of vapor formation during steady-state conditions irradiation (kg m^−2^ h^−1^), and *Q* denotes the intensity of the incoming solar irradiation (mW cm^−2^). *H*_e_ is the result of adding the sensible and latent heat enthalpies, which is calculated as follows:

3 *H*_e_ = *C*_p_Δ*T* + Δ*H*

where, *C*_p_ is water's specific heat capacity (4.18 J g^−1^ k^−1^), Δ*T* is water's temperature change, and Δ*H* is the latent heat of vaporization (2394 kJ kg^−1^) on the relative temperature.

### 2.11. Water solar desalination artificial seawater and wastewater test

The preparation of the artificial seawater sample, which contains NaCl (1000 ppm), MgSO_4_·7H_2_O (1500 ppm), and CaCl_2_ (1000 ppm), involved dissolving deionized water in saltwater that had undergone solar desalination. By using data from inductively coupled plasma optical emission spectroscopy, we showed the concentrations of the principal ions (Na^+^, Mg^2+^, and Ca^2+^) in the saltwater. In order to obtain polluted wastewater, 10 ppm of methylene blue and rhodamine B were also prepared.

### 2.12. Solar steam generation recycling test

Ten solar steam generation cycles were performed to assess the reusability of the Ag-PMG nanocomposite foam. Using the solar simulator system, the samples were exposed to 1 kW m^2^ of light for each cycle. The values of water mass loss and the surface temperature were calculated after one hour in 1 sun. The wetted polyurethane foam was cleaned, dried, and then reused for the following cycle.

## 3. The antibacterial activity

The antibacterial activities of the fabricated samples were tested against Gram-positive *B. subtilis* and Gram-negative *E. coli* bacteria by agar disc diffusion technique using inoculums containing 106 bacterial spread on Muller–Hinton agar plates. The sample discs were placed on the surface of the agar plates seeded with the test organisms. The plates were incubated at 37 °C. Diameters of inhibition zones (mm) were measured after 18–24 hours for bacteria.

## 4. Result and discussion

### 4.1. Characterization

**Fig. 1:**
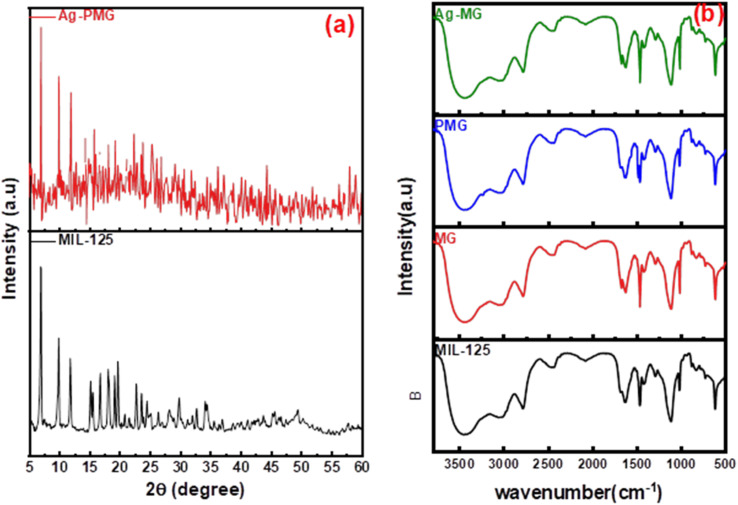
(a) XRD patterns and (b) FTIR spectra of pure and modified MIL-125.

Fig. 1b shows the XRD patterns of the synthetic composites and pure MIL-125. The XRD pattern of pure MIL-125 has prominent peaks that were in excellent agreement with our previously published literature, and demonstrate the good crystallinity of the frameworks.^54^ The MIL structure with 3D pores was created and connected to the sharing of μ-OH corners with TiO_6_ octahedral interconnected to linker (H_2_BDC) molecules. For pure MIL-125, the peaks allocated at 2*θ* = 6.9°, 9.9°, 11.9°, 15.7°, 18°, 19.2°, 22.8°, 23.7°, 34.3° and 49.3° are assigned to the (101), (002), (211), (220), (310), (222), (312), (213), (400) crystal planes, respectively.^55–58^ This result demonstrated that MIL-125 crystals were completely formed under the influence of GO sheets.^59^ The XRD pattern of the presence of polyaniline in the composite illustrated the same crystal structure of MIL-125 during the fabrication of the composite.^60^ There are apparent weak diffraction peaks at 38.1° and 44.2° in the Ag-MG composite, indicating that Ag is loaded on the composite's surface,^61^ so the Ag peaks were magnified and are labeled in Fig. S1† to show the diffraction peak.

The functional groups of all fabricated composites were observed by using FTIR spectra, as depicted in Fig. 1a. The significant broad adsorption band in the 3200–3400 cm^−1^ range was shown in the FTIR spectra of MIL-125 for free solvent molecules in the framework pores.^62^ The bands at 1291–1690 cm^−1^ were produced by the vibration of carboxylate groups due to the linker in the frameworks.^63^ The characteristic absorption band of the benzene ring was shown in the 800–1200 cm^−1^ range, while the bands at 500–800 cm^−1^ belonged to Ti–O–C and Ti–O–Ti vibrations.^64^ The absorption band increased around 3400 cm^−1^ and 1600 cm^−1^ due to the O–H vibration and Ti–O–C bonds, respectively, owing to the interactions between MIL-125 and GO nanosheets.^65^ A new absorption band around 1000 cm^−1^ was assigned as the π–π interaction between the frameworks and GO sheets. In addition, the absorption band originating from the graphene skeletal vibration was observed at 1626 cm^−1^. New bands at 1125 and 805 cm^−1^ belonged to the –C–N stretching and –C–H out-of-plane bonding in the benzenoid ring, respectively.^66,67^ In addition, an absorption band was observed at 3251 cm^−1^ due to the amine group stretching.^68,69^ The band intensity decreased compared to MG due to π–π interaction between PANI with graphene oxide, and hydrogen bonding between them. The peak at 1627 cm^−1^ exhibited low intensity compared to MG due to AgNPs on the graphene oxide surface.

**Fig. 2:**
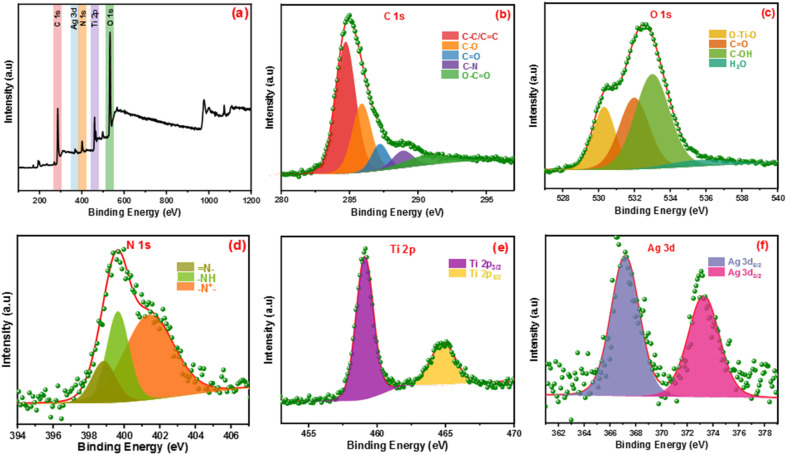
(a) Full survey XPS spectrum of Ag-PMG, (b–f) high-resolution XPS of C 1s, O 1s, N 1s, Ag 3d and Ti 2p, respectively.

The elemental states and chemical compositions of all composites have been estimated using HR-XPS analysis. Fig. 2 shows the XPS of the Ag-PMG composite that determined the existence of C 1s, O 1s, N 1s, Ti 2p and Ag 3d elements. HR-XPS of the C 1s pattern was deconvoluted into five peaks at 284.69, 285.92, 287.21, 288.92 and 290.83 eV, attributed to C–C (sp^2^, sp^3^), C–N, C=O, and –COOR, respectively.^70–72^ The O 1s spectrum was divided into three main peaks at 530.28, 531.97, 532.96 and 535.93 eV that were ascribed to metal–oxygen bonds (O–Ti–O), –OH in the organic ligand and oxygen adsorbed at the surface (C=O) and H_2_O molecules, respectively.^73^ The N 1s spectrum was divided into three peaks at 401.36, 399.64 and 398.80 eV binding energies, which belonged to the (–N^+^), quinoid imine bonds (–NH–) and benzenoid amine bonds (=N–)of polyaniline, respectively.^74,75^ The peaks at 459.09 and 464.75 eV resulted from the deconvolution of the Ti 2p spectrum to two peaks belonging to Ti 2p_1/2_ and Ti 2p_3/2_, respectively.^76^ The Ag 3d spectrum exhibited two peaks assigned to Ag 3d_3/2_ and Ag 3d_5/2_, and the spin-orbital photoelectron peaks are shown at 367.20 and 373.23 eV, respectively.^77,78^

**Fig. 3:**
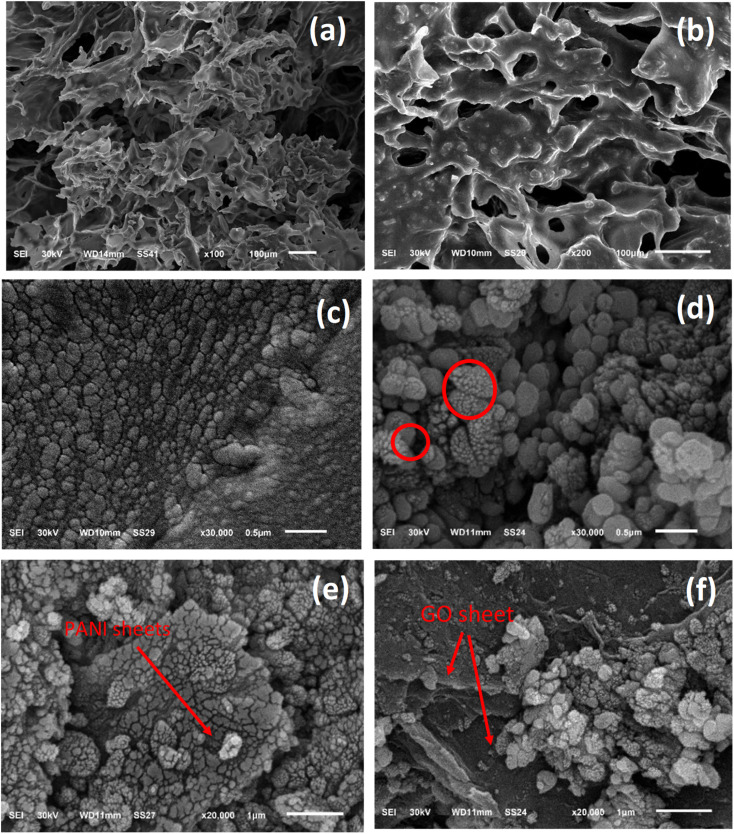
SEM images of (a) PU foam, (b and c) Ag-PMG loaded on PU foam, (d–f) Ag-PMG composite.

The morphology of the prepared composite was studied by SEM analysis before and after loading on the PU foam. Fig. 3 displays the structural information of the Ag-PMG composite, as determined by SEM. The high porosity of the polyurethane foam is shown in the SEM images, which contain the open porous, hydrophilic nature and cellular morphology of the PU matrix. The characteristics of the PU foam are important for water supply from the porous surface with low heat loss to bulk water for solar evaporation.^79–81^ MIL-125 features a spherical shape with a smooth surface. From the SEM images, it can be seen that GO and PANI have a sheet-like shape. PANI was uniformly dispersed throughout MG and exhibited a deep layer with a pore structure, as well as numerous fold structures and agglomerations on the layer's surface. With the addition of Ag NPs, the surface of the material becomes rough and randomly deposited on the surface of PMG.^82–85^ Moreover, the surface area was measured for pristine MIL-125, which shows a typical type (I) curve with a high surface area of 1120 m^2^ g^−1^. Loading the sample with PANI and Ag nanoparticles decreased the surface area to 560 m^2^ g^−1^ without affecting the porosity of the prepared material (Fig. S2†).

### 4.2. Solar steam generation process

To determine the steam generation performance for various fabricated photothermal absorbers, five polyurethane foams with a diameter of 4.5 cm were made using the following samples: M, MG, Ag-MG, PMG and Ag-PMG. All photothermal absorbers were demonstrated and contrasted by using polyurethane foam in 1 sun over 1 h by the solar simulator for the solar steam generation performance. Each sample was mixed with polyurethane foam with 4.5 cm inner diameter. The foam was moistened on the surface of a volume of 100 mL water in a beaker, and all were wetted in deionized water before putting on the water surface. The mass loss was calculated by using an electronic balance, and the evaporation rate was measured from the mass loss was determined at 5 min up to 1 h.

**Fig. 4:**
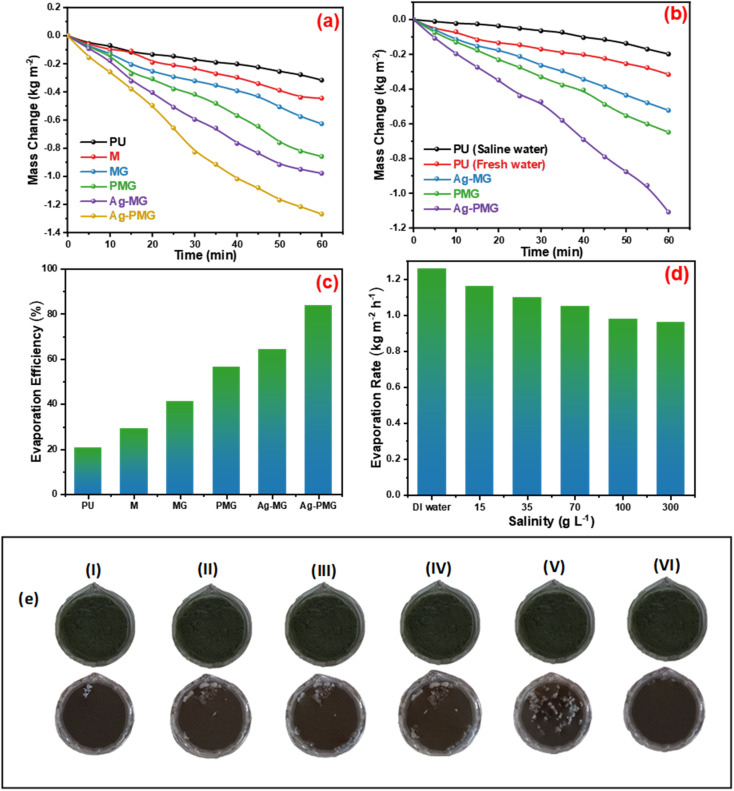
(a) Mass changes for PU, M, MG, PMG, Ag-MG and Ag-PMG. (b) Mass change for Ag-MG, PMG and Ag-PMG of pure and saline water. (c) The evaporation efficiency of fabricated foams. (d) The evaporation rate of Ag-PMG for various concentrations of saline for 60 min, and (e) salt-rejection performance during a daytime salt aggregation time from (I) 5 h to (V) 9 h, and (VI) light-off salt diffusion times.

**Fig. 5:**
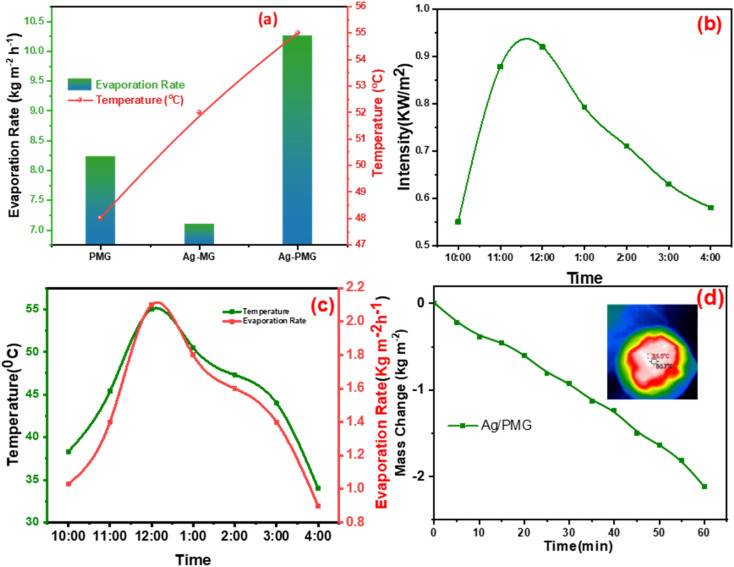
Outdoor solar water evaporation system in real sunlight. (a) Evaporation rate during 7 h of outdoor solar desalination recorded over time from 10:00 to 4:00, and temperature of PMG, Ag-MG and Ag-PMG at high intensities. (b) Solar radiation intensities measured over time from 10:00 to 4:00. (c) Evaporation rate and temperature of Ag-PMG recorded over time from 10:00 to 4:00. (d) The mass change of Ag-PMG under the real sun for 60 min, and IR images of Ag-PMG at a high intensity of outdoor solar desalination.

The mass loss of five photothermal absorbers of the pure water (polyurethane foam) from the steam generation test is depicted in Fig. 4a. The results show that pure PU foam has the smallest mass loss of water of 0.31 kg m^−2^ between another sample. This is because pure PU foam has a low thermal absorptivity, but also moved the light absorption by its modification on the surface foam.^86,87^ The mass change of the (M, MG, PMG, Ag-MG and Ag-PMG) absorbers are 0.44, 0.26, 0.85, 0.97 and 1.26 kg m^−2^, respectively, which demonstrated the combination of the frameworks with GO and plasmonic nanoparticles. MIL-125 was known as a photosensitive material and has a three-dimensional pore structure from phthalic acid and Ti–O octahedron. MIL-125 is a porous material with highly ordered crystal structures from organic linkers and metal ions with large active sites, which lead to excellent photocatalytic efficiency, water permeability and salt rejection in the desalination process. MIL-125 has good electron–hole separation performance because of its Ti–O clusters with isolated titanium oxide quantum dots.^88,89^ The solar absorbers including GO enhanced the rate of evaporation because of transmittance loss, the low thermal conductivity, and low reflectance of GO.^90^ The hydroxyl and carboxylic functional groups in the graphene oxide structure were important for enhancing the evaporating water because the strong π–π interactions between the MOF and GO can effectively increase the electron–hole pair separation and enhance the photocatalytic performance. The solar absorbers containing PANI increased the evaporation rate and the mass change due to good light absorption properties, chemical stability and high conductivity for solar-heating interfacial evaporation, unique photothermal conversion capability and good adhesive properties.^91,92^ The solar absorbers containing silver nanoparticles in Ag-MG and Ag-PMG increased the rate of evaporation in contrast to other absorbers because of their efficient plasmonic activity in the visible light region and broad light absorption band by the plasmonic nanoparticles. After the sample was prepared, the results demonstrated that the Ag-PMG composite has a high mass loss of 1.26 kg m^−2^ because of the incorporation of MIL-125, GO, PANI and Ag NPs. Fig. 4b shows the results of photothermal foams in the artificial saline water for PU, PMG, Ag-MG and Ag-PMG evaporators. The mass losses of the absorbers are 0.19, 0.65, 0.52 and 1.1 kg m^−2^, respectively. In Fig. 4b, the mass loss in saline water was lower than water because of the presence of salt ions. The mass loss decreased by increasing the amount of salt in the solution. In Ag-MG and PMG, the mass change and evaporation rate decreased for saline water because salt on the foam's surface had precipitated. It blocked incident light and reduced water evaporation. The mass loss in the presence of PANI was more than that in the presence of Ag (Ag-MG) because PANI has the ability to reject the salt ions, and prevented the active sites of the solar absorber from being blocked.^93,94^ This layer of accumulated salts could cause a small reduction in the evaporation rate. We can show the effect of salt rejection by using PMG and Ag-MG composite in saline water (30 wt%). Salt crystallization forms in the evaporation, and leads to the formation of salts on the surface of foams. The aggregated salt should be removed, dissolved back, or collected by additional techniques. Natural dissolution is an easy technique to remove salts from the surface foam, and then extract salt from brine. We examined the salt-rejection performance of fabricated foam. Fig. 4e shows the dynamics of salt accumulation and rejection during a 9 hours period. PMG exhibited higher salt-rejection capability than Ag-MG during the test due to the salt rejection capacity of PANI. In the desalination process, the positive charges of the benzenoid and quinonoid structures and the electrostatic interaction of the functional groups of PANI with ions of salt caused the “Donnan exclusion” effect, so PMG has high broadband absorption efficiency. Salt crystals did not form in the first four hours because of the large solubility of salt in water and the high diffusive transport of salt in foam. After four hours, salt started to form and accumulate on the PU surface. The accumulation of salt increased with time during evaporation. After radiation was switched off, the salt on the evaporator started to dissolve back into the bulk brine after a long time, showing salt-rejection performance.^95–98^ In Fig. 4c, the solar evaporation performance was shown by evaporation efficiency and the evaporation rate under 1 sun. It was illustrated that the Ag-PMG nanocomposite has a high evaporation rate and efficiency that reached 1.26 kg m^−2^ h^−1^ and 90%, respectively, which is considered as one of the largest values shown for different photothermal materials demonstrated for solar steam generation, as proved by the data showed in Table 1. Fig. 4d shows the evaporation rate of the Ag-PMG composite, which was performed under different concentrations of NaCl (1.5%, 3.5%, 7%, 10% and 30%) under 1 sun irradiation for 60 min, showing a small change in mass loss. The stability of the evaporation rates at different concentrations of saline water was connected to the salt rejection effect of PANI in the sample, and the evaporation rate increased due to plasmonic behavior. The outdoor tests were performed under natural sunlight for 7 h from 10:00 a.m. to 4:00 p.m. The practical performance of Ag-MG, PMG, and Ag-PMG was noticed by recording the change of intensity of the solar light, the mass loss of artificial saline water, and the temperature of the photo absorber system. The natural sunlight intensity was the largest at 1:00 p.m and is equivalent to 920 W m^−2^, and the sunlight intensity increased over time until it reached 920 W m^−2^, then deceased as shown in Fig. 5a. The rate of evaporation reached 7.10, 8.23 and 10.26 kg m^−2^ after 7 h under continuous irradiation of real sunlight for the thermal evaporators PMG, Ag-MG and Ag-PMG, respectively, as shown in Fig. 5b. The change in the surface temperature and the evaporation rate for the outdoor experiments showed that the temperature reached 55 °C at 1:00 p.m. for Ag-PMG, as shown in Fig. 5c. The mass loss was measured at time intervals of 5 min up to 1 h, as shown in Fig. 5d. Moreover, the results confirmed that the Ag-PMG photoabsorber system exhibited excellent photothermal performance and a high potential for generation of clear water.

**Table 1:** Solar steam generation performances of different photothermal materials under 1 sun illumination

Evaporator	Support	Solar intensity (kW cm^−2^)	Evaporation rate (kg m^−2^ h^−1^)	Efficiency (%)	Reference
Ag/Au-GO	PU	1	1.00	63.0	79
Fe_2_O_3_-rGO	—	1	1.12	70.0	99
Modified graphene aerogel	—	1	1.2	76.9	100
Ag@TiO_2_ nanoparticles	A flexible filter membrane	1	0.9	68.6	101
C–Au-TiO_2_	Glass or polished Cu	1	0.97	68	102
rGO polyurathene nanocomposite foam	PU	1	1	65	103
Ag_2_S@PANI/carbonaceous porous material	PU	1	1.1	91.5	104
NiCo_*x*_S_*y*_-PANI@GF	Glass fiber membrane	1	1.3	78.7	105
Acid-doped polyaniline	Polystyrene foam	1	1.3	80.7	106
rGO/TiO_2_ composite	—	1	1.2	—	107
Ag/PANI/GO@MIL-125	PU	1	1.26	90	This work

**Fig. 6:**
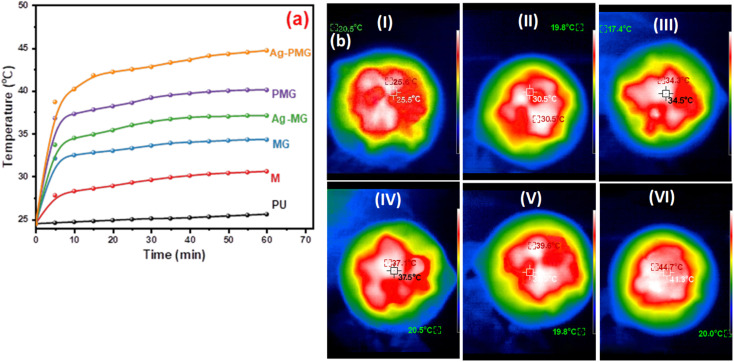
(a) Temperature change on foam surfaces for different composites in 1 hour, and (b) IR thermal images of (I) PU, (II) M, (III) MG, (IV) PMG, (V) Ag-MG, and (VI) Ag-PMG after 1 h.

Solar steam generation for the different fabricated photothermal absorbers, PU, M, MG, PMG, Ag-MG and Ag-PMG, compared the temperature change of various solar absorbers after 1 sun irradiation for 1 h, as shown in Fig. 6a. The temperature on the top surface was rapidly enhanced from room temperature to 25.5, 30.6, 34.3, 37.1, 39.6 and 44.7 °C, respectively. The Ag-PMG temperature was the largest because of the hydrophilic surface property and high solar absorption due to Ag, PANI and GO together. After radiation for 1 h, the temperature changes on the foam surfaces were measured by IR camera and thermocouple, as depicted in Fig. 6b.

**Fig. 7:**
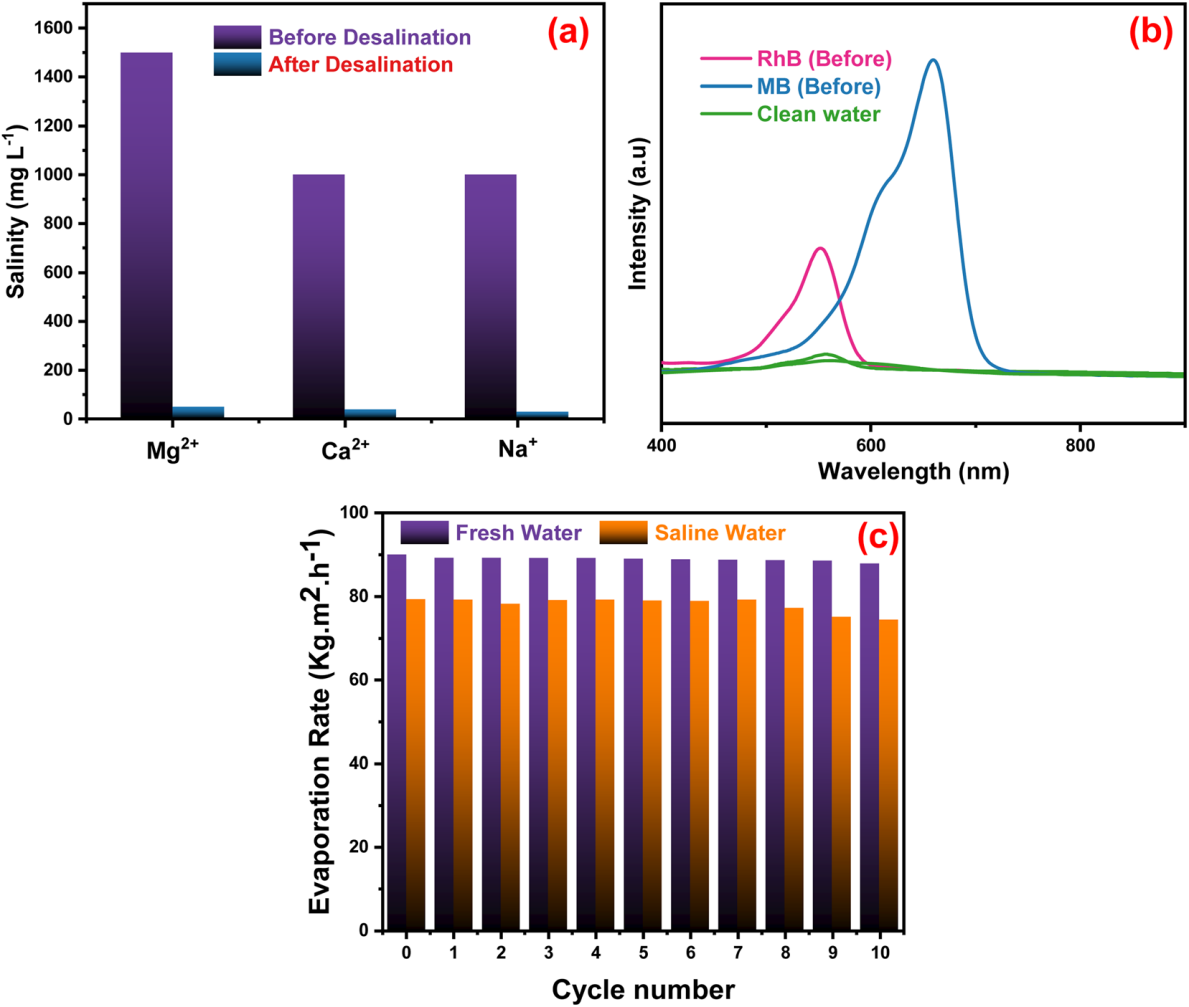
(a) Concentration of metal ions before and after the desalination technique by using Ag-PMG composite, (b) UV-vis absorption spectra of dye-contaminated water before and after desalination, and (c) reusability test of the Ag-PMG sample during 10 cycles.

The quality of the evaporators was assessed in Fig. 7a when artificial seawater was present, and a condensing unit was used to collect the evaporated water. ICP-OES was used to measure the concentrations of Mg^2+^, Ca^2+^ and Na^+^ ions in the condensed water. The concentration of the three ions decreased significantly after desalination with the Ag-PMG evaporator. The ion concentrations decreased dramatically from their first levels of 1500, 1000, and 1000 mg L^−1^ of Mg^2+^, Ca^2+^ and Na^+^, respectively, which are below the standard values set by the World Health Organization for drinking water.^108^ The synthesized system was investigated using excellent desalination techniques compared to common techniques. The Ag-PMG composite can be used to purify the contaminated water with RhB and MB dyes. Fig. 7b shows the absorption spectra of the polluted water before and after desalination, and demonstrated that all dyes have been eliminated from the evaporated water. Fig. 7c shows the reusability and stability of the Ag-PMG photoabsorber in seawater desalination by using 10 cycles of evaporated experiments in artificial seawater (1 hours of radiation for each cycle). The high efficiency of Ag-PMG after 10 cycles with a slight decrease can be attributed to plasmonic Ag nanoparticles and salt rejection of PANI, which prevent the blocking of the active sites of the solar absorber.^24^

## 5. M, PMG and Ag-PMG composite tests as antibacterial agents

**Fig. 8:**
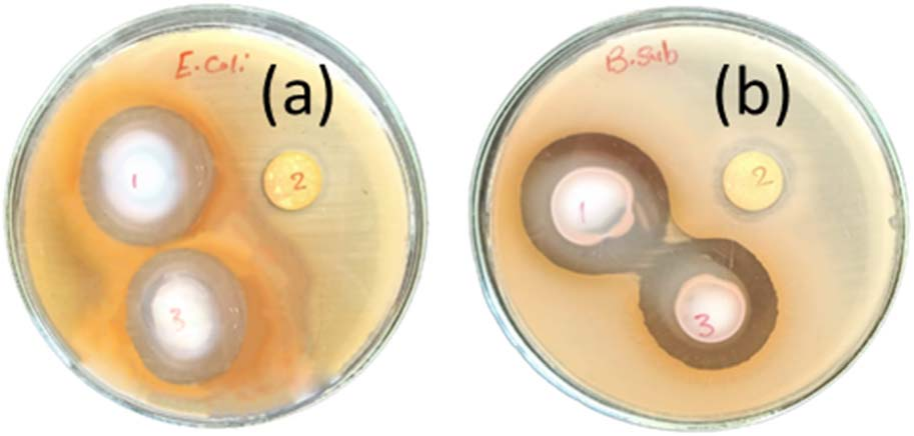
Antibacterial activity tests of (1) Ag-PMG, (2) M, (3) PMG composites against both (a) *E. coli* and (b) *B. subtilis* bacteria.

Antibacterial activity tests of M, PMG and Ag-PMG composites against both Gram-positive *B. subtilis* and Gram-negative *E. coli* bacteria were performed, and are shown in Fig. 8 and Table 2. MIL-125 has activity against *B. subtilis* but not against *E. coli* bacteria because the Gram-positive type gives more negatively charged surfaces than the Gram-negative type, and MIL-125 has antibacterial activity against *B. subtilis* by producing a concentration of Ti^4+^ as an agent to produce positive charges. Its positive charges of Ti react with the negative charge cell membrane, affecting the structure of the cell membrane and changes its permeability, which leads to the death of the bacteria. GO has antibacterial activities due to the hydrophobic interaction with the membrane phospholipids. Ag-PMG composites have the highest antibacterial activity against both Gram-positive and Gram-negative bacteria by the inhibition zone compared to the PMG and M composites because of the electrostatic adsorption between Ag and PANI molecules and bacteria cells, and the presence of silver nanoparticles kills bacteria. Ag reacts with the cytoplasmic membranes of the bacteria, leading to bacterial inactivation and death. Ag's spherical particles can easily enter cells through membrane holes, and the composite may shatter inside the cell due to reactions with sulphur and phosphorous-containing DNA and protein contents. The presence of silver ions bound to bacterial DNA disturbs bacterial respiration and adenosine triphosphate synthesis. Polyaniline and Ag have powerful antibacterial effects on bacterial strains. Also, it demonstrated stronger antibacterial defenses against the Gram-negative cell membrane by lowering the osmotic pressure, which supports their physical connection. Moreover, the increased permeability of the bacterial cell wall and the formation of stress-resistant biofilms are caused by the numerous chemical properties of polyaniline because of the surface hydrophilicity and the polymer chain length. The charged functional groups in the composite have an increased reaction with the bacteria cell. The inhibition zones of the studied bacteria are in the following order: *B. subtilis* > *E. coli*. This represents more activity of Ag and PANI on *B. subtilis* than on *E. coli*.^48,109–112^

**Table 2:** Antibacterial activity of M, PMG and Ag-PMG nanocomposites

Sample	*B. subtilis*	*E. coli*
	Inhibition zone (mm)	Inhibition zone (mm)
M	15	−ve
PMG	31.6	28.1
Ag-PMG	33.3	30.3

## 6. Conclusion

In summary, we prepared a new system of graphene/MIL-125 polyurethane (MGPU) nanocomposite foam for solar water desalination. The system has low thermal conductivity, porous structure, high absorption and the capacity for self-floating. MIL-125 has a pore structure, large active sites, water permeability, high surface area, and salt rejection in the desalination process. We can modify the MGPU foam by using plasmonic Ag and polyaniline. PANI improved the salt rejection capability and Ag improved the solar photothermal energy conversion in water desalination. The solar conversion efficiency of the Ag-PMG composite reaches 90% during 1 sun irradiation, and the evaporation rate is 1.26 kg m^−2^ h^−1^ connected to the physical properties of MIL-125 and GO, PANI and Ag. As a result, for continuous evaporation of 10 h, the evaporator showed stable evaporation. We showed the difference between the two composites, including PANI and Ag, and the effect of salt rejection in the composite. We also noticed that the composite has good durability after more than 10 cycles with no significant reduction in performance activity. The fabricated composites can eliminate dyes from contaminated water and produce clean condensed freshwater. The presence of rGO, PANI and Ag in the fabricated catalysts enhances the antimicrobial activity.

## Conflicts of interest

The authors declare no conflict of interest.

## Supplementary Material

RA-013-D3RA02242K-s001
