# Supplementary material for: Solar-driven seawater desalination via plasmonic hybrid MOF/polymer and its antibacterial activity
Source: RSC Adv. 2023 Jun 20;13(27):18525–37. doi: 10.1039/d3ra02242k (PMC10280044; doi:10.1039/d3ra02242k)
Supplement: RA-013-D3RA02242K-s001 [file RA-013-D3RA02242K-s001.pdf]

Electronic Supplementary Material (ESI) for RSC Advances.
This journal is © The Royal Society of Chemistry 2023

**Solar-driven seawater desalination via plasmonic hybrid MOF/ polymer and its anti-bacterial activity.**

**Ola. R. Hayes, Amr Awad Ibrahim*, Mina Shawky Adly, S. E. Samra, A. M. A. Ouf, S. A. El-Hakam, Awad I. Ahmed ***

*Chemistry Department, Faculty of Science, Mansoura University, Al-Mansoura 35516, Egypt.*

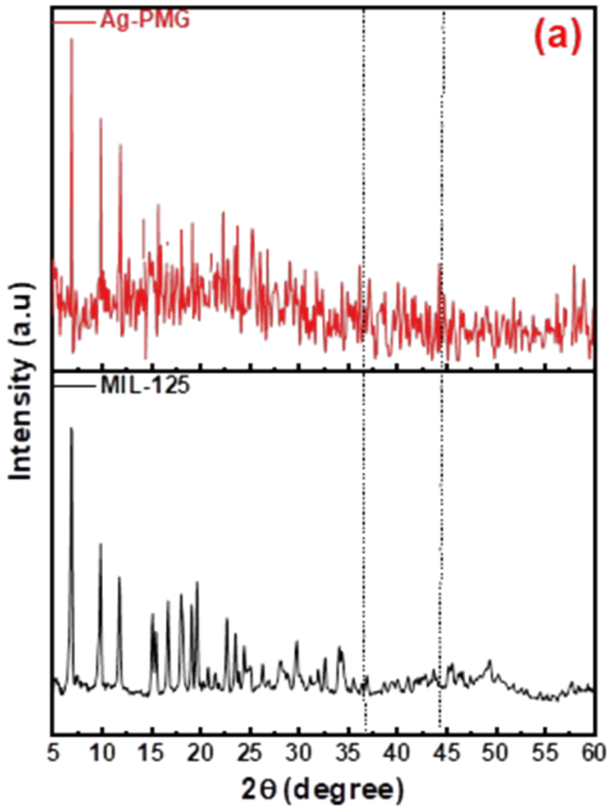


Fig. 1S. (a) XRD patterns of MIL-125 and modified MIL-125 and (b) XRD of Ag peak

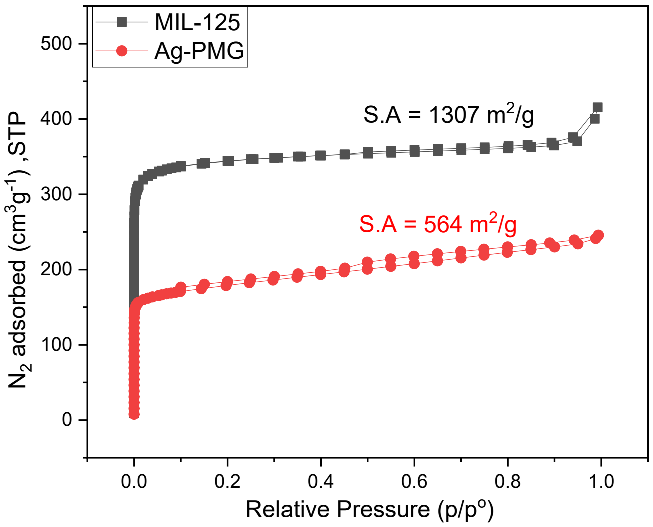


Fig. 2S. $N_2$ adsorption-desorption isotherms for MIL-125 and Ag-PMG
